# Toward in vivo proof of binding of ^18^F-labeled inhibitor [^18^F]TRACK to peripheral tropomyosin receptor kinases

**DOI:** 10.1186/s13550-022-00915-w

**Published:** 2022-07-30

**Authors:** Melinda Wuest, Justin J. Bailey, Jennifer Dufour, Darryl Glubrecht, Vanessa Omana, Tom H. Johnston, Jonathan M. Brotchie, Ralf Schirrmacher

**Affiliations:** 1 Department of Oncology, Cross Cancer Institute, University of Alberta, 11560 University Ave, Edmonton, AB T6G 1Z2 Canada; 2 The Neuro - Montreal Neurological Institute-Hospital, McGill University, Montreal, QC Canada; 3 Krembil Research Institute, University Health Network, Toronto, ON Canada; 4 Atuka Inc., Toronto, ON Canada; 5 Department of Oncology, Medical Isotope Cyclotron Facility, University of Alberta, 6820-116 St, South Campus, Edmonton, AB T6H 2V8 Canada

**Keywords:** TrkA, TrkB, PET, Molecular imaging, Radiotracer, [^18^F]TRACK

## Abstract

**Background:**

Tropomyosin receptor kinases (TrkA, TrkB, TrkC) are a family of tyrosine kinases primarily expressed in neuronal cells of the brain. Identification of oncogenic alterations in Trk expression as a driver in multiple tumor types has increased interest in their role in human cancers. Recently, first- and second-generation ^11^C and ^18^F-labeled Trk inhibitors, e.g., [^18^F]TRACK, have been developed. The goal of the present study was to analyze the direct interaction of [^18^F]TRACK with peripheral Trk receptors in vivo to prove its specificity for use as a functional imaging probe.

**Methods:**

In vitro uptake and competition experiments were carried out using the colorectal cancer cell line KM12. Dynamic PET experiments were performed with [^18^F]TRACK, either alone or in the presence of amitriptyline, an activator of Trk, entrectinib, a Trk inhibitor, or unlabeled reference compound TRACK in KM12 tumor-bearing athymic nude mice as well as B6129SF2/J and corresponding B6;129S2-*Ntrk2*^*tm1Bbd*^/J mice. Western blot and immunohistochemistry experiments were done with KM12 tumors, brown adipose tissue (BAT), and brain tissue samples.

**Results:**

Uptake of [^18^F]TRACK was increasing over time reaching 208 ± 72% radioactivity per mg protein (*n* = 6/2) after 60 min incubation time. Entrectinib and TRACK competitively blocked [^18^F]TRACK uptake in vitro (IC_50_ 30.9 ± 3.6 and 29.4 ± 9.4 nM; both *n* = 6/2). [^18^F]TRACK showed uptake into KM12 tumors (SUV_mean,60 min_ 0.43 ± 0.03; *n* = 6). Tumor-to-muscle ratio reached 0.9 (60 min) and 1.2 (120 min). In TrkB expressing BAT, [^18^F]TRACK uptake reached SUV_mean,60 min_ 1.32 ± 0.08 (*n* = 7). Activation of Trk through amitriptyline resulted in a significant radioactivity increase of 21% in KM12 tumor (SUV_mean,60 min_ from 0.53 ± 0.01 to 0.43 ± 0.03; *n* = 6; *p* < 0.05) and of 21% in BAT (SUV_mean,60 min_ from 1.32 ± 0.08; *n* = 5 to 1.59 ± 0.07; *n* = 6; *p* < 0.05) respectively. Immunohistochemistry showed TrkB > TrkA expression on BAT fat cells, but TrkA > TrkB in whole brain. WB analysis showed sevenfold higher TrkB expression in BAT versus KM12 tumor tissue.

**Conclusion:**

The present data show that radiotracer [^18^F]TRACK can target peripheral Trk receptors in human KM12 colon cancer as well as brown adipose tissue as confirmed through in vitro and in vivo blocking experiments. Higher TrkB versus TrkA protein expression was detected in brown adipose tissue of mice confirming a peripheral functional role of brain-derived neurotrophic factor in adipose tissue.

**Supplementary Information:**

The online version contains supplementary material available at 10.1186/s13550-022-00915-w.

## Background

Tropomyosin receptor kinases (TrkA, TrkB, TrkC) encoded as *NTRK1, NTRK2*, and *NTRK3* represent a family of tyrosine kinases mainly expressed in the neuronal cells of the brain. They consist of an extracellular transmembrane region and an intracellular section containing the tyrosine kinase domain [1, 2]. Extracellular regions of each Trk subtype bind a preferred neurotransmitter: nerve growth factor (NGF) on TrkA, brain-derived neurotrophic factor (BDNF), and neurotrophin-4 on TrkB and neurotrophin-3 on TrkC [3–9]. The intracellular domain endogenously binds ATP [10, 11].

Trk receptors play an important role during prenatal development of central and peripheral nervous system and normal brain function [10, 12]. Neurotrophin activation of Trk has an impact on neuronal events such as neuronal cell differentiation and survival, cell proliferation, synaptic formation and plasticity, membrane trafficking, and axon and dendrite formation [2, 13, 14]. Neurodegenerative diseases such as Alzheimer’s and Parkinson’s induce changes in Trk expression and signaling [15–19].

Identification of oncogenic alterations in peripheral Trk expression has stimulated interest in their role in human cancers [1]. Unrestrained activation of Trk-dependent pathways resulting from Trk fusion proteins leads to cell transformation, growth, and proliferation [1, 2, 20, 21]. However, Trk fusions are rare and found in only < 1% of solid tumors [21]. A recent immunohistochemical patient analysis found Trk fusions in only 31 (0.3%) out of 11,502 patients [22]. Other studies detected TrkA fusions in 3.3% lung cancers, 0.5–2% colorectal cancers, < 12% papillary thyroid carcinomas, TrkA/B/C fusions in 7% pediatric gliomas, and TrkC fusions in 92–100% secretory mammary tumors [23, 24]. Targeting Trk fusions represents one of the first examples of a ‘personalized medicine’ approach [25]. Two pan-Trk inhibitors have been recently approved for clinical treatment, larotrectinib [26] and entrectinib [27]. For a patient’s inclusion into a Trk inhibitor therapy, noninvasive tools are needed to determine their Trk fusion expression status. Fluorescence in situ hybridization (FISH), next sequencing generation (NSG), standard RT-PCR, or immunohistochemistry analysis using tumor biopsy specimen collected invasively are known [28–36].

There is a need to develop noninvasive methods to reduce the invasiveness of diagnostic procedures. Radiolabeled probes for positron emission tomography (PET) represent a sensitive noninvasive approach. Over the past years, several ^11^C- and ^18^F-labeled kinase domain-binding Trk inhibitors derived from pan-Trk and TrkA subtype selective lead structures were developed [37]. A first-generation radioligand based on the 4-aza-2-oxindole structure resulted in ^11^C-labeled GW441756 [38]. Due to its isomeric mixture, and observed metabolism and pulmonary retention, it was not further developed [37]. Subsequent radioligands in the first generation had linear elongated structures for better cell membrane penetration. ^18^F-labeled GW2580 and QMICF were kinome selective pan-Trk inhibitors [39, 40]. A second radiotracer generation was developed based on imidazo[1,2-*b*]pyridazine- and pyrazolo[1,5-*a*]pyrimidine-containing structures resulting in ^11^C-labeled IPMICF16 [41]. Compared to the first-generation compounds pure enantiomer *(R)*-IPMICF16 resulted in higher potencies (IC_50_) against Trk: 0.2 nM (TrkB), 0.1 nM (TrkC), 4.0 nM (TrkA) [42]. In vivo evaluation in mice, rats, non-human primates and ‘first-in-human’ showed moderate brain uptake. [^11^C]-*(R)*-IPMICF16 is a substrate for active P-glycoprotein 1 efflux [42]. Non-human and human PET data revealed no efflux from the brain and enriched accumulation in thalamus > cerebellum and cortex.

To overcome disadvantages of ^11^C (half-life 20 min), ^18^F-labeled pan-Trk inhibitor TRACK was developed representing an ^18^F-labeled analogue of *(R)*-IPMICF16 [43]. In vitro potency toward all three Trk subtypes was similar to [^11^C]-*(R)*-IPMICF16. Non-human and ‘first-in-human’ PET imaging revealed slightly higher uptake in the cerebellum, thalamus, and cortex [44].

Based on the complex transmembrane protein structure of Trk, its activation and dimerization status as *NTRK* fusion protein, and the localization of the intracellular ligand-binding sites, it remains challenging to demonstrate ‘proof-of-target’ binding for these novel radiolabeled Trk inhibitors in vivo. The goal of the present study was to analyze direct interaction of [^18^F]TRACK with peripheral Trk proteins in vivo to prove its specificity for its use as a functional PET imaging probe.

## Methods

### General

All chemicals, reagents, and solvents for synthesis and analysis were analytical grade. If not stated otherwise, all chemicals and reagents were obtained from Sigma-Aldrich (Sigma-Aldrich, Oakville, ON, Canada).

### Radiosynthesis

The radiosynthesis of [^18^F]TRACK was performed similarly as previously reported [43, 44] utilizing alcohol-enhanced copper-mediated radiofluorination [45]. In brief, no carrier-added aqueous [^18^F]fluoride was produced by an ^18^O(p,n)^18^F nuclear reaction through the bombardment of an [^18^O]H_2_O target. Aqueous [^18^F]fluoride was passed through the female end of a QMA Sep Pak Light Carbonate (46 mg) cartridge (1.6–1.9 GBq in 1 mL H_2_O). The trapped [^18^F]fluoride was pre-dried by-passing *n*-butanol (3 mL) through the cartridge, followed by air (10 mL). [^18^F]fluoride was eluted from the cartridge directly into a glass V-vial containing the boronic ester precursor (4.8 mg, 9.1 μmol) by passing a solution of Et_4_NHCO_3_ (3.5–4.0 mg) in *n*-butanol (0.4 mL) through the male end of the cartridge, with a routine elution efficiency of 75–80%. The reaction vial, with no vial cap, was placed in a 110 °C oil bath for 20 min. The vial was removed from the oil bath and the reaction solution diluted with HPLC eluent (0.5 mL) and drawn up in a syringe. The reaction solution was injected on HPLC (eluent (isocratic): 55% MeCN in H_2_O; flow rate: 3 mL/min, injection loop: 1 mL, column: Phenomenex Luna 3 μm PFP(2) 100 Å, 250 × 10 mm). The peak eluting at ~ 21 min was collected and diluted with H_2_O (15 mL) and passed through a C18 Sep Pak Light cartridge, followed by H_2_O (5 mL) and air (10 mL). [^18^F]TRACK was eluted from the cartridge with ethanol (10 drops). The volume was reduced to ~ 20 μL under an N_2_ stream at 90 °C to afford 112–140 MBq of [^18^F]TRACK for injection.

### Cell culture

Human colon adenocarcinoma KM12 cells were obtained from Charles River Laboratories, National Cancer Institute, Frederick National Laboratory Cancer Research (Fort Detrick, Frederick, MD, USA.), and cultivated in RPMI 1640 cell growth medium supplemented with 10% fetal bovine serum (FBS) (Gibco) and 2 mM L-glutamine (Sigma-Aldrich). For the in vitro cell experiments, cells were seeded in 12-well plates in their medium and grown for 24 h.

### In vitro* cell uptake experiments*

Right before the experiment, the media was removed and the cells were washed two times with phosphate-buffered saline solution (PBS). Next, 200 µL Krebs–Ringer buffer (120 mM NaCl, 4 mM KCl, 1.2 mM KH_2_PO_4_, 2.5 mM MgSO_4_, 25 mM NaHCO3, 70 µM CaCl_2_, 5 mM glucose pH 7.4) was added to each well. Next 300 µL Krebs–Ringer buffer with 0.1–0.5 MBq [^18^F]TRACK radiotracer was added to each well, and the plates were incubated at 37 °C for specific periods of time (1, 5, 10, 15, 30, 45, and 60 min). Radiotracer uptake was stopped with 1 mL ice-cold PBS, and the cells were washed twice with PBS and lysed in 0.4 mL radioimmunoprecipitation assay buffer (RIPA buffer). Radioactivity in the cell lysates was measured as counts per minute [CPM] using a WIZARD2 Automatic gamma counter (PerkinElmer; Waltham, MA, USA) and converted to the radioactivity dose SI unit Becquerel [Bq]. Total protein concentration in the samples was determined by the bicinchoninic acid method (BCA; Pierce, Thermo Scientific 23,227) using bovine serum albumin (BSA) as protein standard. Data were normalized as percent of measured radioactivity per mg protein (%radioactivity / mg protein). Graphs displaying cell uptake over time were constructed using GraphPad Prism 5.0 (GraphPad Software, San Diego, Ca, USA).

Competition binding experiments were carried out in the presence of increasing concentrations of reference compound TRACK (10^−8^ – 10^−7^ M) or pan-Trk inhibitor entrectinib (10^−8^ – 10^−6^ M; Tocris Biosciences, Bristol, UK). Blocking compounds were added to the cells immediately before radiotracer [^18^F]TRACK was applied and incubated for 60 min. After incubation cells were rinsed with ice-cold PBS, lysed, and counted as described above to obtain CPM values. Data then were normalized as % maximum of total added radioactivity from control wells not receiving any blocker compound. Competition binding curves were also generated using GraphPad Prism 5.0 (GraphPad Software, San Diego, Ca, USA).

### Animal experiments

Female athymic nude mice (10–12 weeks old) were received from Charles River Laboratories (Saint-Constant, QC, Canada) and injected subcutaneously with 3 × 10^6^ KM12 cells in 100 µL PBS/Matrigel (50:50) into the upper left flank. After about 3 to 4 weeks, subcutaneous KM12 tumors reached sizes of ~ 400–500 mm^3^ suitable for PET imaging experiments. Female B6129SF2/J (wild-type control) and female B6129S2-*Ntrk2*^*tm1Bbd*^/J (TrkB 50% knockout) mice were obtained from Jackson Laboratories (JAX™ Bar Harbor, ME, USA) at the age of 10–12 weeks and used over the next 3–5 months.

### Dynamic PET imaging

General anesthesia of wild-type control or tumor-bearing mice was induced with inhalation of isoflurane in 40% oxygen/60% nitrogen (gas flow = 1 ml/min), and mice were subsequently fixed in prone position. The body temperature was kept constant at 37 °C for the entire experiment. Mice were positioned in a prone position into the center of the field of view. A transmission scan for attenuation correction was not acquired. Radioactivity present in the injection solution (0.5 mL syringe) was determined using dose calibrator (AtomlabTM 300, Biodex Medical Systems, New York, USA). After emission scan was started, radioactivity (3–8 MBq in 100–150µL saline) was injected with a delay of ~ 15 s through a tail vein catheter. For blocking experiments, 5 mg/kg entrectinib (pan-Trk inhibitor) was pre-injected 15 min before the radiotracer or 0.1–0.5 mg/kg unlabeled reference compound TRACK was co-injected i.v. together with the radiotracer. Pre-dosing with 15 mg/kg amitriptyline was done i.p. 4 h before the radiotracer was injected. PET data acquisition was performed in 3D list mode for 60 min. Dynamic list mode data were sorted into sinograms with 54 time frames (10 × 2 s, 8 × 5 s, 6 × 10 s, 6 × 20 s, 8 × 60 s, 10 × 120 s, 5 × 300 s). Frames were reconstructed using ordered subset expectation maximization (OSEM) or maximum a posteriori (MAP) reconstruction mode. No correction for partial volume effects was performed. Image files were further processed using the ROVER v2.0.51 software (ABX GmbH, Radeberg, Germany). Masks defining 3D regions of interest (ROI) were set and defined by 50% thresholding. Mean standardized uptake values [SUV_mean_ = (activity/mL tissue)/(injected activity/body weight), mL/kg] were calculated for each ROI. Time–activity curves (TAC) were generated from the dynamic scans.

### Western blotting

Tissue samples were homogenized on ice using a sonicator using RIPA buffer with Triton-X-100 (RB4478, Biobasic, Markham, ON, Canada). The protein concentrations were determined using Pierce™ BCA Protein Assay Kit (Thermo Scientific, Waltham, MA, USA). Samples were loaded onto a 4–12% precast polyacrylamide gel (Bio-Rad, Hercules, CA, USA), separated by SDS-PAGE, and transferred to PVDF membranes. The membranes were first air-dried for 1 h and re-hydrated with 100% methanol. The membranes were blocked with 5% non-fat powdered milk and then incubated with a combination of either rabbit monoclonal anti-TrkA antibody (ab76291; Abcam, Cambridge, UK; 1:1000) and mouse monoclonal anti-TrkB [MM0586-7Y6] (ab89925; Abcam, Cambridge, UK; 1:1000) or rabbit polyclonal anti-TrkB (ab18987; Abcam, Cambridge, UK; 1:10,000) and mouse monoclonal anti-TrkA antibody (ab86474; Abcam, Cambridge, UK; 1:1000) and rabbit monoclonal anti-Actin antibody (A2066, Sigma-Aldrich, St. Louis, MI, USA, 1:10,000) overnight at 4 °C. The membranes were incubated with IRDye 680RD goat anti-rabbit (92,668,071, LI-COR, Lincoln, NE, USA, 1:15,000) and IRDye 800CW goat anti-mouse (92,532,210, LI-COR, Lincoln, NE, USA, 1:15,000). Protein bands were visualized using Odyssey Infrared-Imaging System and analyzed using Empiria Studio Software (LI-COR, Lincoln, NE, USA).

### Immunohistochemistry for TrkA and TrkB

Fresh samples of brown adipose tissue, KM12 tumor tissue, and whole brain were fixed in neutral-buffered 10% formalin overnight and embedded into paraffin. Slides with 4 μm sections were dried at 60 °C for 1 h and re-hydrated by 3 changes of xylene for 10 min each and then graded ethanol from 100 to 50%, followed by water and TBS. Slides were microwaved in a pressure cooker for 6 min in citraconic anhydride (0.05% in water, pH 7.4) for antigen retrieval and blocked with 0.5% fish gelatine in Tris-buffered saline with 0.05% Tween-20 (TBST) for 30 min and incubated with rabbit monoclonal anti-TrkA antibody [EP1058Y] (ab76291; Abcam, Cambridge, UK; 1:50) or rabbit polyclonal anti-TrkB antibody (ab18987; Abcam, Cambridge, UK; 1:50) in a humidity chamber overnight at 4 °C. After incubation in 3% H_2_O_2_ in water for 15 min, slides were incubated with DakoCytomation Envision + anti-mouse- or anti-rabbit-labeled Polymer HRP (DakoCytomation, Glostrup, Denmark) for 1 h, developed, using Dako Liquid DAB + Substrate Chromagen System and 1% copper sulfate and counterstained with hematoxylin. Slides were dehydrated by reversing re-hydration and cover slipped. Quantification analysis of protein expression was conducted with ImageJ 1.x (NIH and LOCI, University of Wisconsin; USA) and IHC profiler [46].

### Data analysis

All data are expressed as means ± standard error of the mean (SEM) from three or more experiments. All graphs were constructed using GraphPad Prism 5.0 (GraphPad Software, San Diego, CA, USA). IC_50_ values were calculated using a nonlinear regression (curve fit) analysis (GraphPad Prism 5.0). Statistical differences were considered significant if p < 0.05 and were evaluated using the Student’s t test.

## Results

**Fig. 1:**
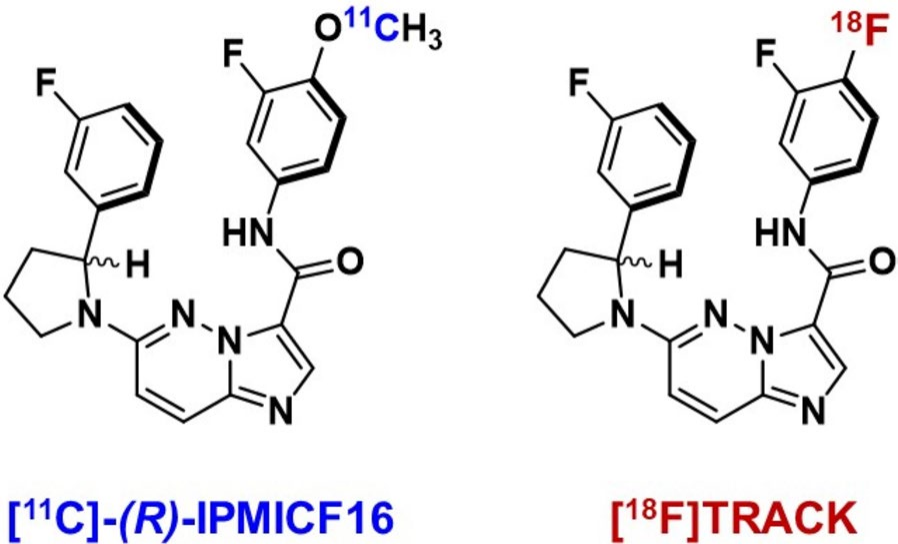
Chemical structures of ^11^C-labeled PET radiotracer *(R)*-IPMICF16 and ^18^F-labeled TRACK

Figure 1 shows the chemical structure of [^18^F]TRACK in comparison with the ^11^C-labeled derivative. Radiolabeling occurred at the same molecule position.

**Fig. 2:**
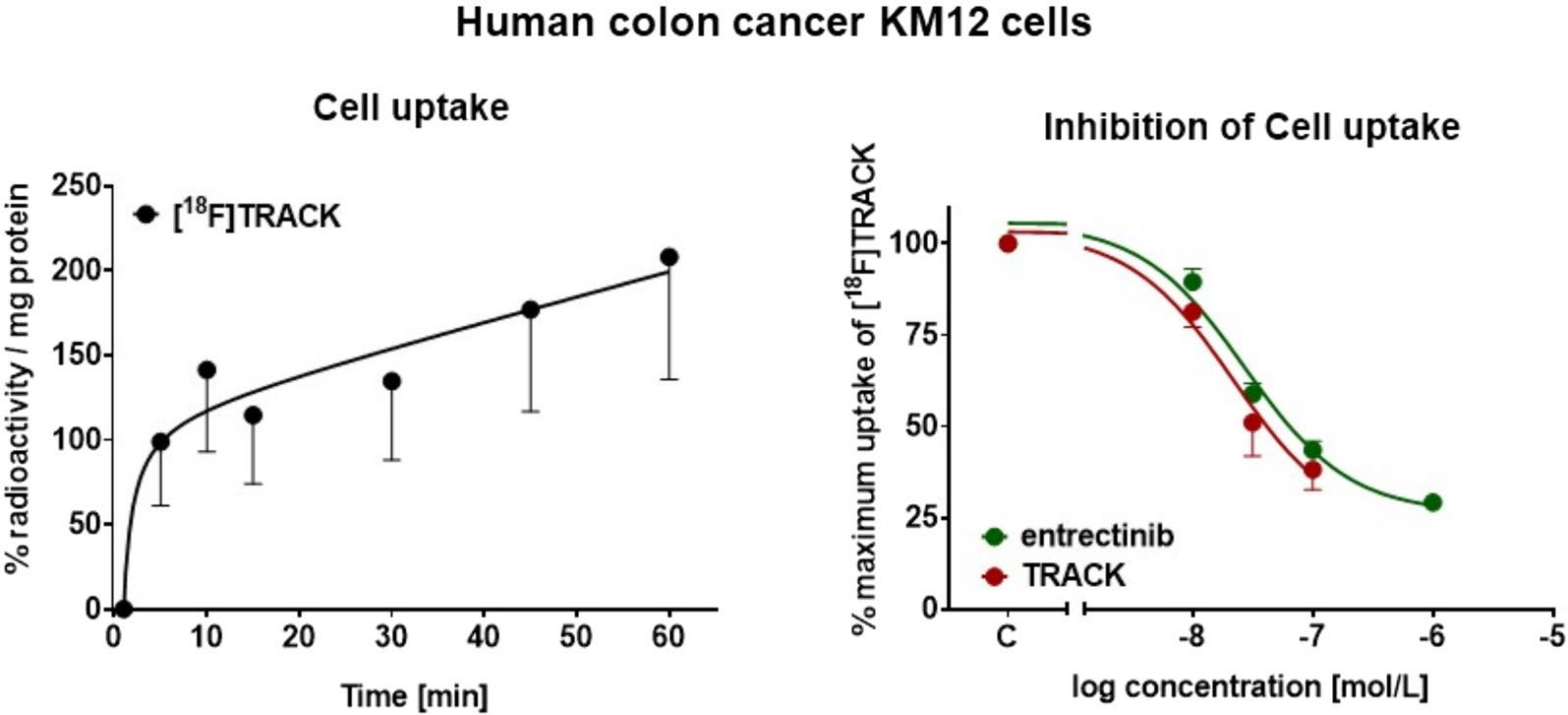
Left) Uptake of [^18^F]TRACK into KM12 cells over time. Data are normalized as % of total radioactivity per mg protein. Right) Inhibitory effect of increasing concentrations of TRACK or entrectinib on cell uptake of [^18^F]TRACK in KM12 cells. Data are normalized as % maximum uptake of [^18^F]TRACK with no inhibitory compound present. C—control at 100% radiotracer uptake. All data are shown as mean ± SEM from 6 analyzed wells out of 2 separate experiments (6/2)

In vitro cell uptake of [^18^F]TRACK was analyzed in human KM12 colon cancer cells. These cells express TrkA, specifically tropomyosin 3-TrkA fusion protein (Vaishnavi et al. 2013; Tatematsu et al. 2014). Uptake of [^18^F]TRACK was increasing over time reaching 208 ± 72% radioactivity per mg protein (n = 6/2) after 60 min incubation time (Fig. 2). Blocking of TrkA was measured trough competitive binding using increasing concentrations of pan-Trk inhibitor entrectinib and compared to the effects of the non-radioactive reference compound TRACK. Similar half-maximum inhibition concentrations (IC_50_) values were determined: 29.4 ± 9.4 nM for TRACK and 30.9 ± 3.6 nM for entrectinib (both n = 6/2), respectively, confirming that radiotracer [^18^F]TRACK was indeed binding to TrkA in vitro and that both compounds, TRACK and entrectinib, functionally blocked TrkA in the analyzed KM12 cells.

**Fig. 3:**
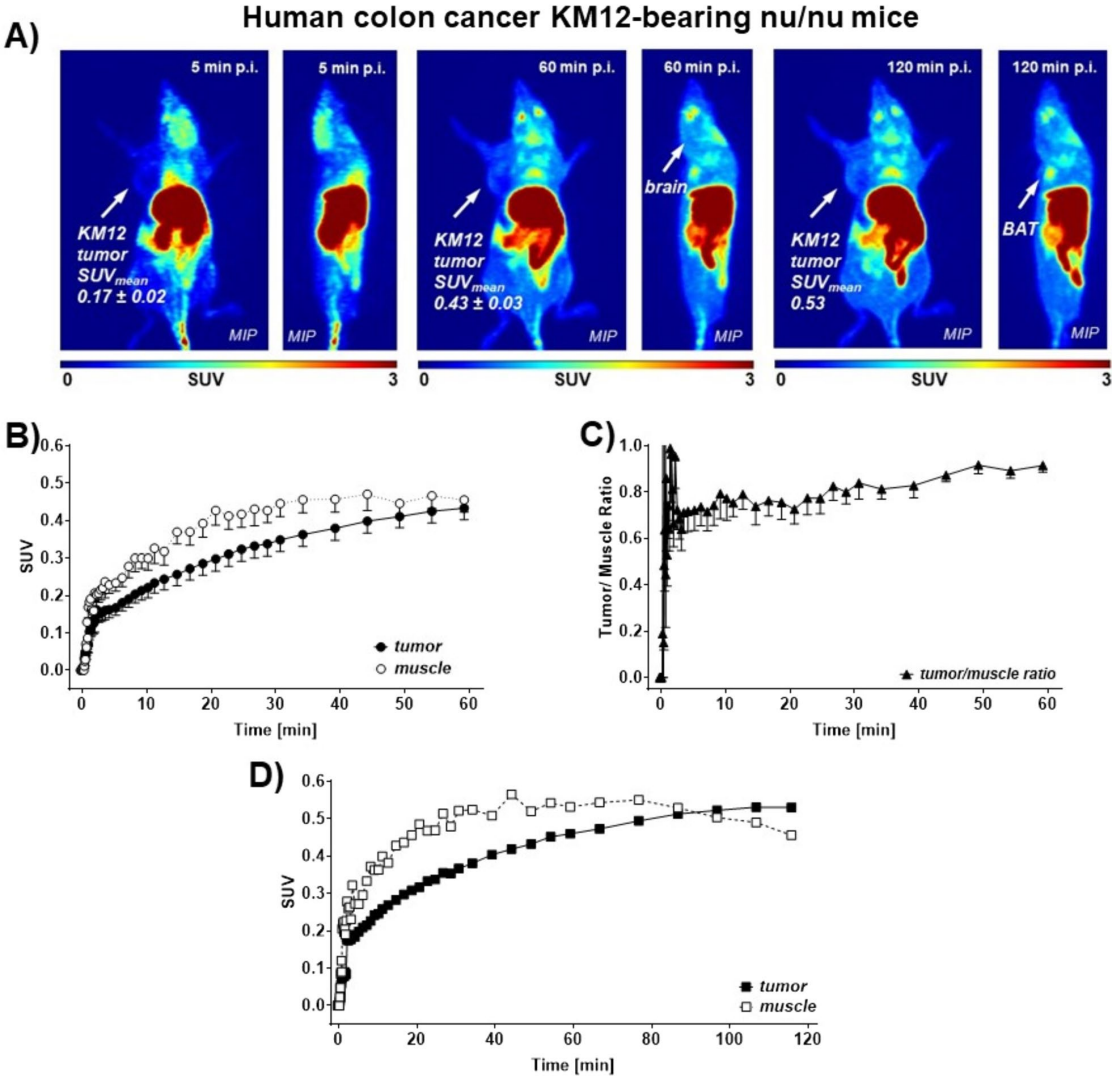
Uptake into KM12 tumor and muscle tissue. **A** Representative PET images as maximum intensity projections (MIP) after 5 min *(left)*, 60 min *(middle)* and 120 min *(right)* post-injection of 5–7 MBq [^18^F]TRACK into a KM12 tumor-bearing athymic nude mouse. **B** Time–activity curves (TACs) for KM12 tumor and muscle tissue over 60 min after injection of [^18^F]TRACK. **C** Calculated tumor-to-muscle ratio over time from the KM12 tumor and muscle tissue data in **B**. **D** TACs for KM12 tumor and muscle tissue over 120 min after injection of [^18^F]TRACK. **C**–**D** All graphical data are shown as mean ± SEM from n experiments. **B** and **C** show data from *n* = 6 experiments; **D** only shows data from n = 2 experiments

The radiolabeled [^18^F]TRACK was also analyzed in vivo in KM12 tumor-bearing mice. Original PET images as well as semiquantitative radioactivity uptake into tumor and muscle tissue are depicted in Fig. 3 (brain uptake in WT B6129SF2/J and B6129S2-*Ntrk2*^tm1Bbd^/J TrkB 50% knockout mice in Additional file 1: Fig. S1). The images show brain uptake and washout over time as well as increasing signal in BAT. The tumor area is visible at 60 and 120 min post-injection, while clearance mainly occurs through the hepatobiliary system as the liver and intestines show high radioactivity levels (Fig. 3A). Tumor uptake was continuously increasing over time from a standardized uptake value (SUV) of 0.22 ± 0.03 at 10 min to 0.43 ± 0.03 (*n* = 6) after 60 min post-injection (Fig. 3B). In contrast, radioactivity in muscle tissue uptake was initially systematically higher with a SUV of 0.30 ± 0.03 at 10 min increasing to 0.46 ± 0.03 (*n* = 6) at 40 min post-injection with no further change up to 60 min post-injection indicative of a beginning washout of the radioactivity. To analyze this further, two KM12 tumor-bearing mice were measured dynamically over 120 min post-injection. While the SUV in the tumor tissue reached 0.53 (*n* = 2) at 120 min in these mice, SUV in muscle tissue amounted to 0.45 after that time frame (*n* = 2; Fig. 3D). Taken together, radioactivity washout from muscle started after ~ 90 min post-injection, while tumor uptake was still increasing. This observation led to the conclusion that accumulating continuous tumor uptake was only occurring after later time points, while uptake into muscle tissue later also resulted in a slow washout process. After 120 min post-injection, tumor-to-muscle ratio (T/M) reached 1.13, while at 60 min it amounted to only 0.9 (Fig. 3C).

**Fig. 4:**
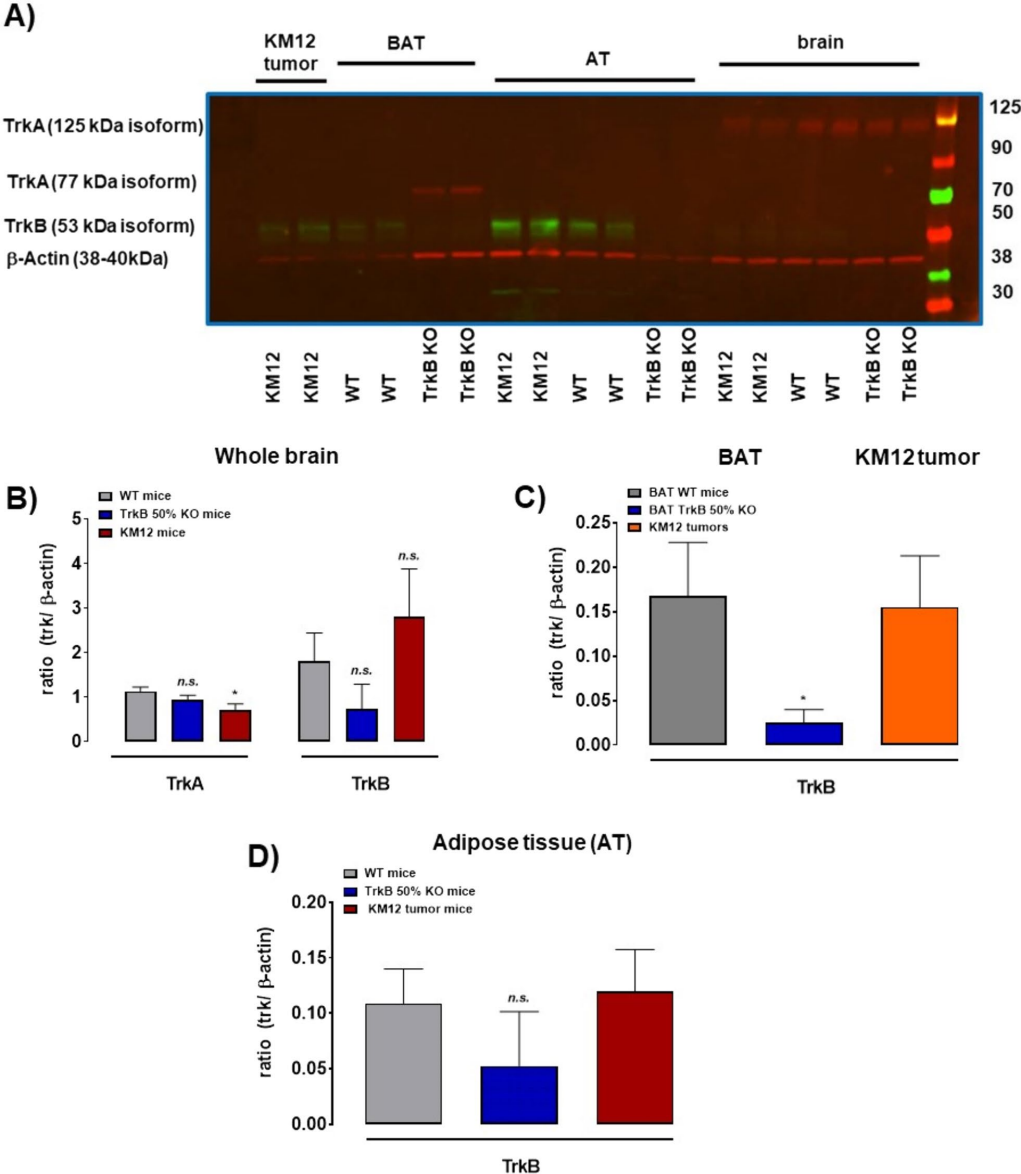
Protein expression of TrkA and TrkB. **A** Original gel of selected samples from KM12 tumor tissue, brown adipose tissue (BAT), white adipose tissue (AT), and the whole brain from KM12 tumor-bearing mice, control wild type (WT), and TrkB 50% KO mice from combined red (700 nm) and green (800 nm) light wavelength analysis showing using antibodies for TrkA and TrkB and β-actin as housekeeping gene. For the full-length gel please, see Additional file 1: Fig. S5. **B** Semiquantitative comparison of TrkA and TrkB protein levels in brain samples from KM12 tumor-bearing mice, control wild type (WT), and TrkB 50% KO mice. **C** Semiquantitative comparison of TrkB protein levels in BAT and KM12 tumor tissue from KM12 tumor-bearing mice, control wild type (WT), and TrkB 50% KO mice. **D** Semiquantitative comparison of TrkB protein levels in white adipose tissue (AT) from KM12 tumor-bearing mice, control wild type (WT), and TrkB 50% KO mice. **B**–**D** All data are shown as normalized ratios (Trk / β-actin) and as mean ± SEM from 5 different tissue samples. * *p* < 0.05; *n.s.*—not significant

Based on the relatively low radioactivity uptake in the peripheral tumors, the next experimental step involved the protein analysis of TrkA and TrkB in brain, BAT, and KM12 tumor tissue of wild type (WT), TrkB 50% KO (B6129SF2/J), and KM12 tumor-bearing athymic mice (Fig. 4). In the whole brain, both TrkA and TrkB proteins were found to be sufficiently expressed with TrkB about twofold–threefold higher than TrkA (Fig. 4B). Expression of TrkB was similar in KM12 tumor tissue and brown adipose tissue (BAT), and 50% knockout of TrkB confirmed reduced protein expression in whole brain and BAT (Fig. 4C). A similar pattern was observed in white adipose tissue (AT) as well (Fig. 4D).

**Fig. 5:**
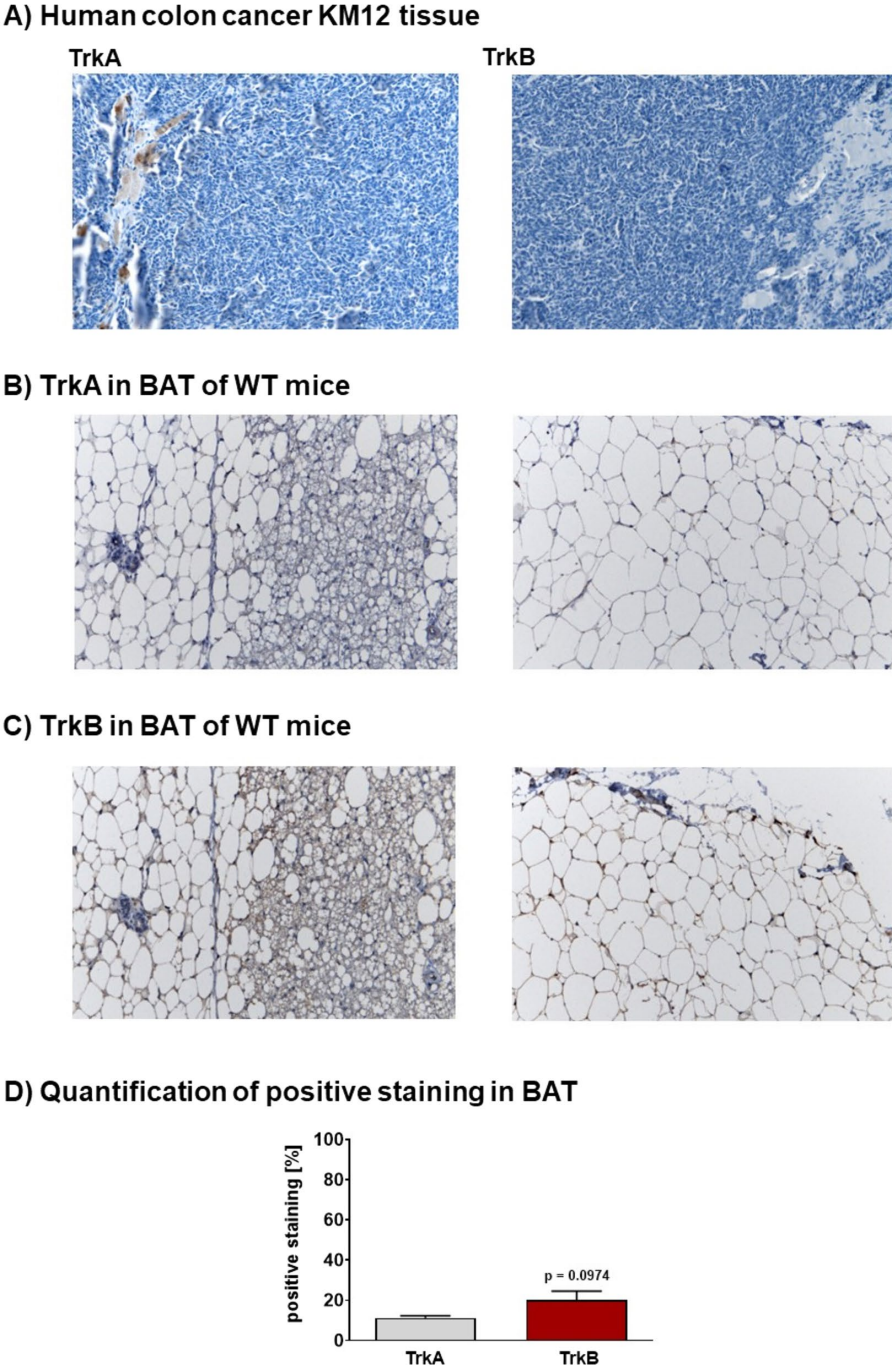
Protein expression of TrkA and TrkB. Immunohistochemical staining of TrkA and TrkB in KM12 tumor tissue samples (**A**) and in BAT of wild-type (WT) control mice (**B**, **C**). Pictures were taken using a 20 × objective. **D** Quantification of positive TrkA and TrkB immunohistochemical staining in BAT. Data as mean ± SEM from *n* = 3 slices

Immunohistochemistry analysis also revealed TrkB protein expression in the membrane of fat cells in brown adipose tissue which was higher than TrkA expression (Fig. 5) as quantified protein positive staining showed: 11.2 ± 1.1% for TrkA versus 20.0 ± 4.7% for TrkB (*n* = 3; *p* = 0.0974; Fig. 5D), respectively. Using the same antibody, no TrkB protein expression was detectable in KM12 tumor tissue as well as TrkA. In the latter sample, only some inclusions into the tumor tissue detected some protein levels (Fig. 5A). For comparison, protein expression of TrkA and TrkB in the brain of KM12 tumor-bearing athymic mice and B6129SF2/J wild-type mice is shown in Additional file 1: Fig. S2.

**Fig. 6:**
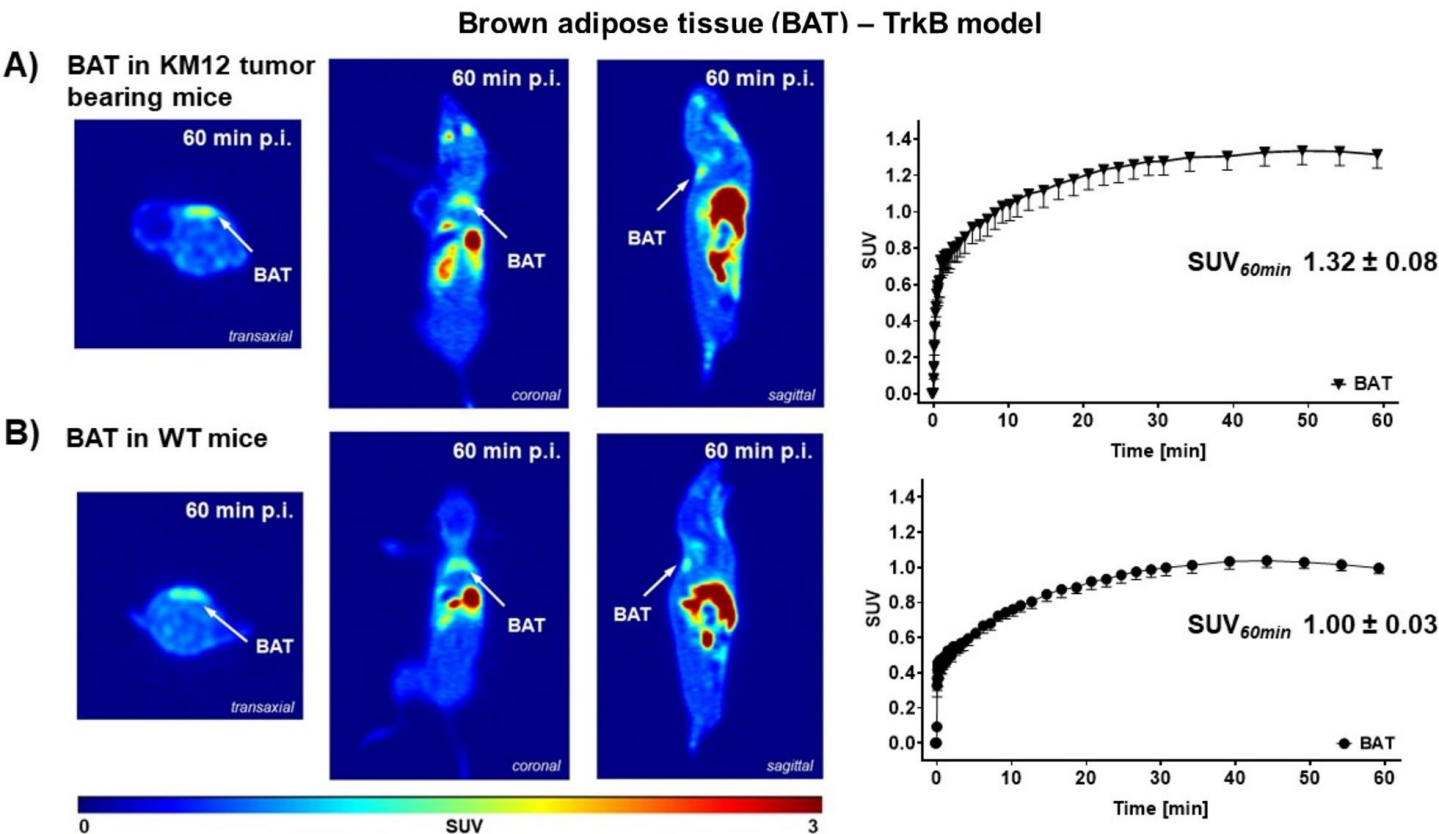
Uptake into BAT tissue. Left) Representative PET images as sagittal projection after 60 min post-injection of 5–7 MBq [^18^F]TRACK into a KM12 tumor-bearing athymic nude mouse (**A**) or wild-type (WT) control mouse (**B**). Right) Corresponding time–activity curves (TACs) for radioactivity profiles in BAT tissue. Data are shown as mean ± SEM from n experiments; upper diagram from *n* = 7 and lower diagram from *n* = 3 different mice

Analysis of dynamic PET data after injection of [^18^F]TRACK resulted in substantially higher radioactivity uptake into brown adipose tissue compared to KM12 tumor tissue before. In KM12 tumor-bearing athymic mice, uptake into BAT resulted in SUV_60min_ 1.32 ± 0.08 (*n* = 7), while in WT mice uptake amounted to 1.00 ± 0.03 (*n* = 3), respectively (Fig. 6). From the dynamic time–activity curves, it was also visible that the uptake levels reached the maximum earlier after ~ 60 min compared to the KM12 tumor uptake (> 2 h). Interestingly, 50% knockout of TrkB in B6129S2-*Ntrk2*^tm1Bbd^/J mice did not lead to a reduced uptake of [^18^F]TRACK in BAT (Additional file 1: Fig. S3).

**Fig. 7:**
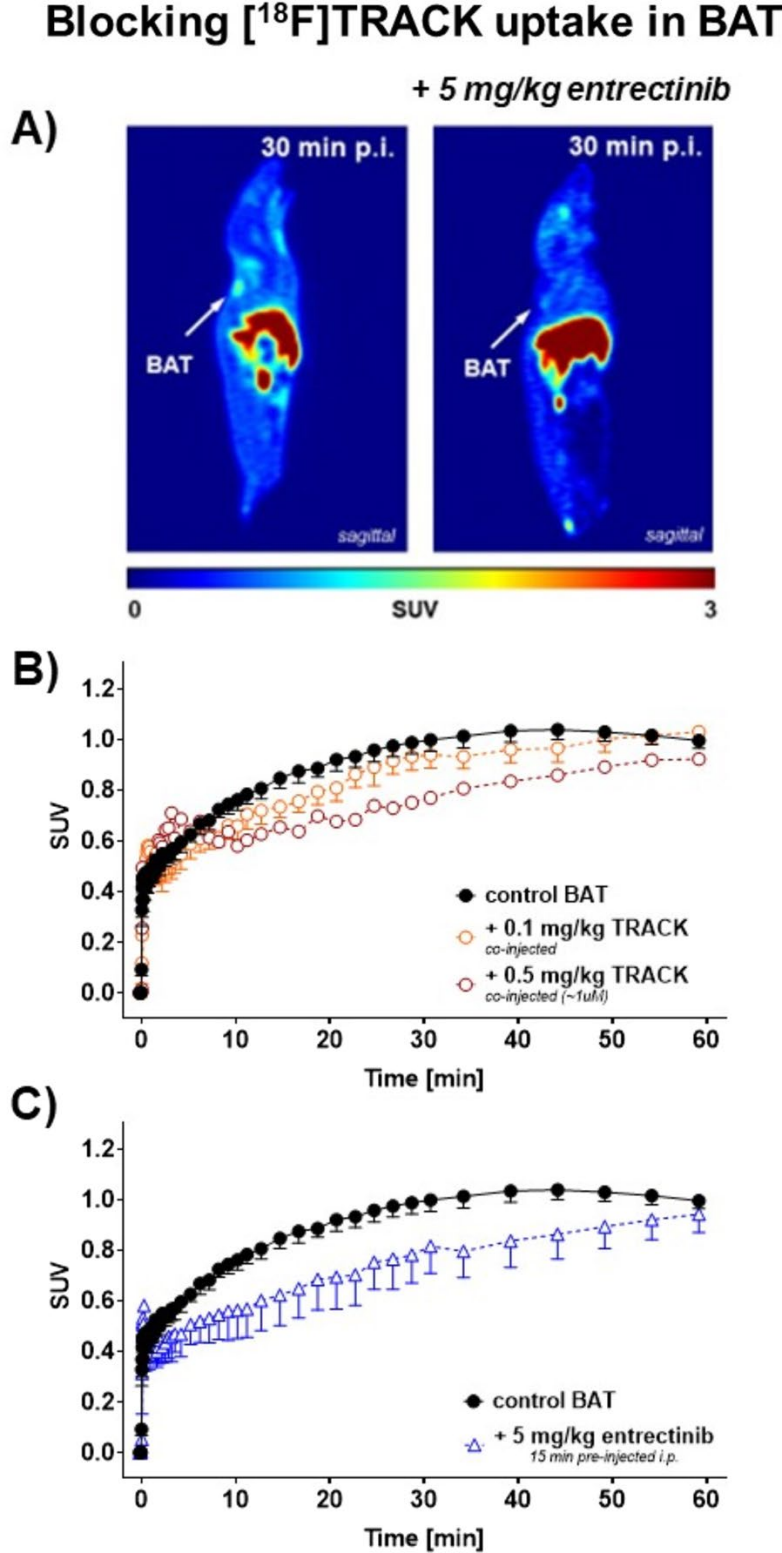
Trk inhibitory effect in BAT tissue. **A** Representative PET images as sagittal projection after 30 min post-injection of 5–7 MBq [^18^F]TRACK into a wild-type (WT) control mouse in the absence *(left)* or presence of 5 mg/kg entrectinib *(right)*. **B** Effects of 0.1 (*n* = 3) and 0.5 mg/kg (*n* = 2) TRACK (co-injected with the radiotracer iv) on the time–activity curve (TAC) of [^18^F]TRACK (*n* = 3) in BAT tissue. **C** Effect of 5 mg/kg (*n* = 3) entrectinib (pre-injected 15 min before the radiotracer i.p.) on the time–activity curve (TAC) of [^18^F]TRACK (*n* = 3) in BAT tissue. **B**–**C** Data are shown as mean ± SEM from n experiments

Specificity of [^18^F]TRACK and its functional binding to Trk in vivo were tested with blocking experiments. Using the non-radioactive reference compound TRACK with doses of 0.1 mg/kg and 0.5 mg/kg co-injected with the radiotracer resulted in a concentration-dependent reduction of the uptake curve in BAT with the highest effects observed at ~ 30 min with a reduction of SUV from 1.00 ± 0.05 (*n* = 3) to 0.77 (*n* = 2) amounting to ~ 23%. Interestingly, this blocking effect was eliminated again at 60 min post-injection (Fig. 7B). In addition to the self-blocking experiment, pan-Trk inhibitor entrectinib was used. A dose of 5 mg/kg pre-injected i.p. 15 min before the radiotracer resulted in a similar blocking pattern: a blocking effect at ~ 30 min post-injection resulting in an SUV of 0.82 ± 0.11 (*n* = 3) corresponding to ~ 18% which was also completely abolished again after 60 min. This was very much like the blocking with TRACK indicating the same functional pattern.

**Fig. 8:**
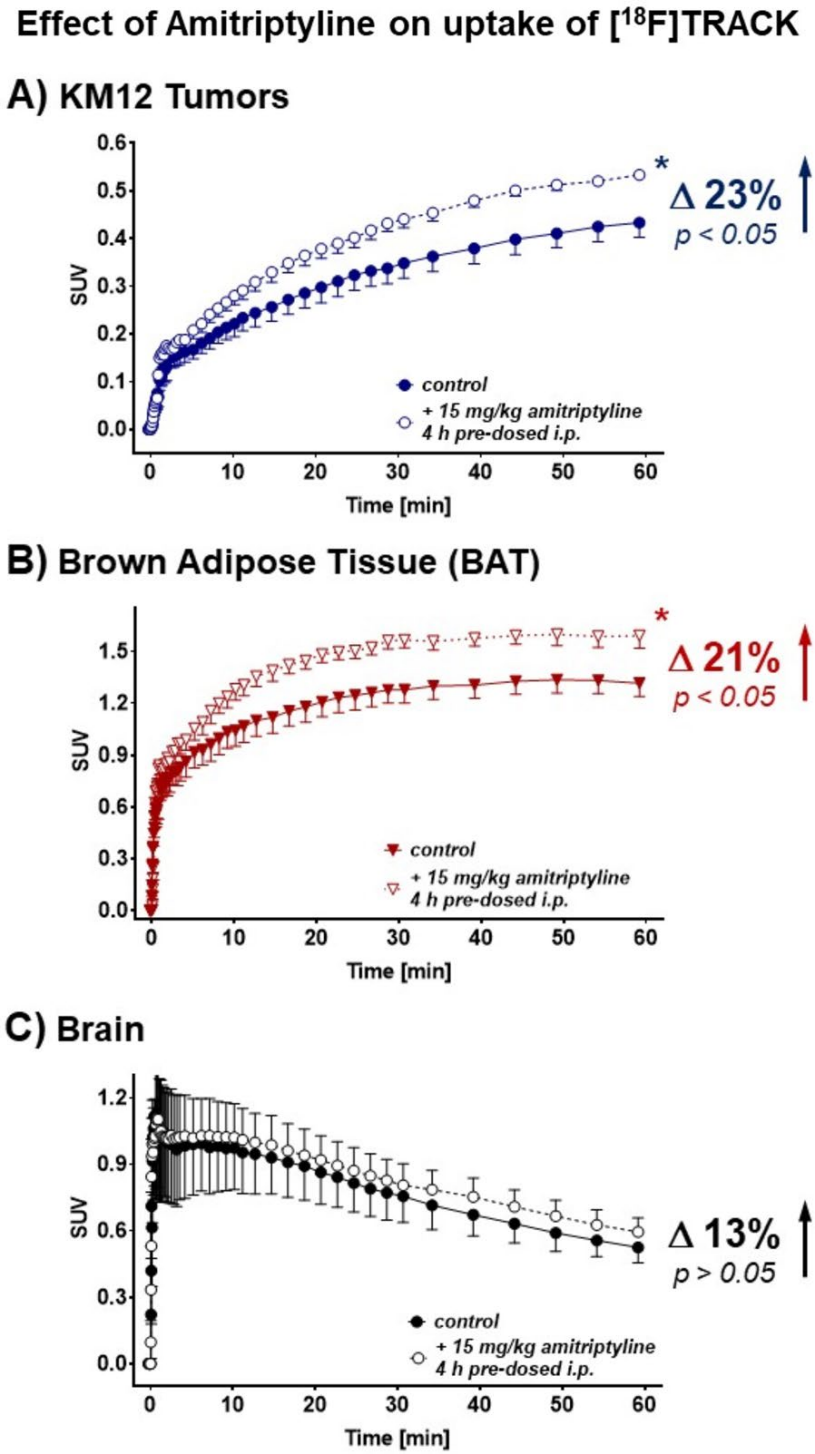
Effect of amitriptyline on uptake of [^18^F]TRACK. Effect of 15 mg/kg amitriptyline (pre-injected 4 h before the radiotracer i.p.) on KM12 tumor uptake (**A**), on uptake into BAT (**B**), and the whole brain (**C**). All data are shown as mean ± SEM from 5–6 experiments

To further prove functional involvement of Trk, the classic tricyclic antidepressant compound amitriptyline was chosen for further in vivo experiments as it was reported to act as a TrkA and TrkB agonist in the literature [47]. Pre-dosing of KM12 tumor-bearing mice with 15 mg/kg amitriptyline 4 h i.p. before radiotracer injection resulted in significant increases in absolute [^18^F]TRACK uptake in KM12 tumor tissue by 23% (SUV_60min_ from 0.53 ± 0.01 to 0.43 ± 0.03; *n* = 6; *p* < 0.05) and also in brown adipose tissue by 21% (SUV_60min_ from 1.32 ± 0.0.08 (*n* = 5) to 1.59 ± 0.07; *n* = 6; *p* < 0.05), respectively (Fig. 8B, C). Effects in the brain were somewhat smaller amounting to an increase of 13% only which was not significant (SUV_60min_ from 0.52 ± 0.07 (*n* = 5) to 0.60 ± 0.06; *n* = 6; *p* > 0.05; Fig. 8C). Taken together, amitriptyline did indeed increase uptake of [^18^F]TRACK in selected peripheral tissue, however, not significantly in the brain. In addition, [^18^F]TRACK uptake into muscle tissue was also increased in the presence of amitriptyline by 15% (SUV_*30min*_ from 0.45 ± 0.03 to 0.52 ± 0.02; *n* = 5; *p* = 0.057; Additional file 1: Fig.  S4). However, at 60 min this difference was diminished which contrasted with the profiles observed in brown adipose tissue and KM12 tumor tissue.

**Fig. 9:**
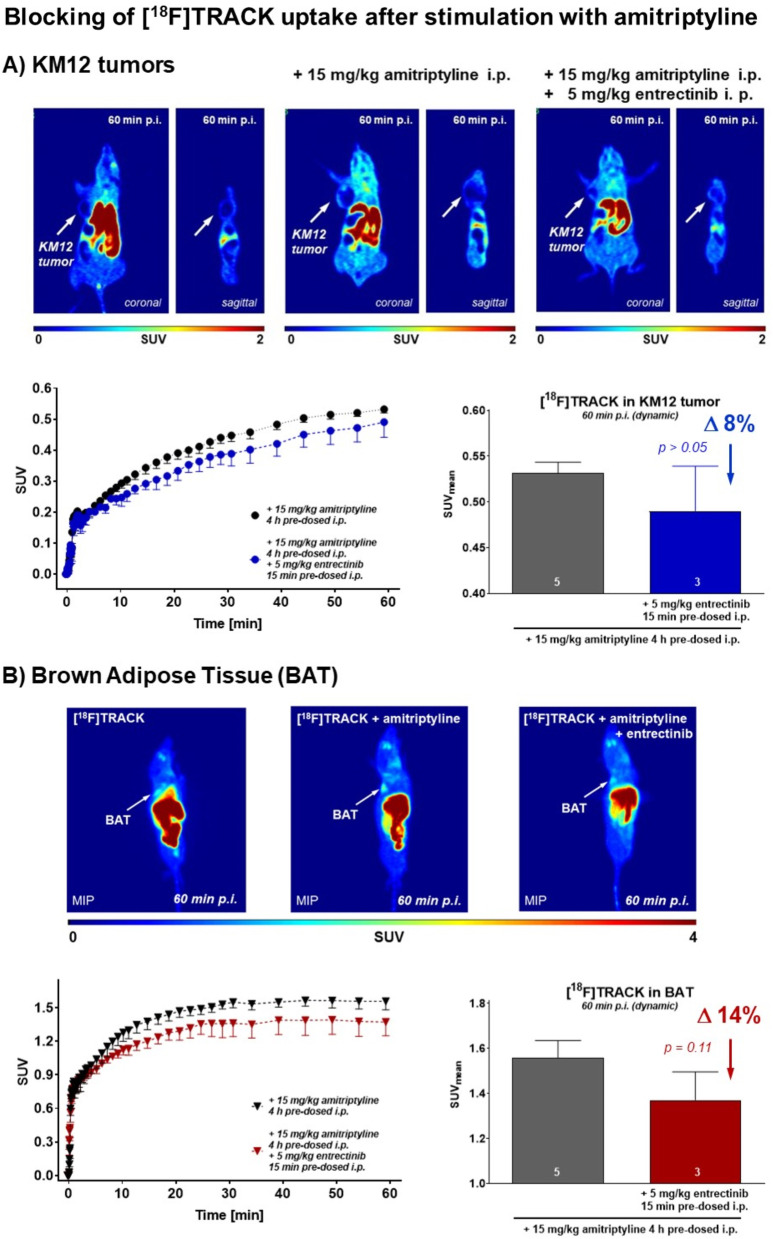
Effect of Trk inhibition in the presence of amitriptyline. **A** Effect on KM12 tumor uptake of [^18^F]TRACK. Representative PET images as coronal and sagittal projections at 60 min after injection of 5–7 MBq [^18^F]TRACK into KM12 tumor-bearing mice. Control image *(left)*, after injection of 15 mg/kg amitriptyline *(middle)* and after combined injection of 15 mg/kg amitriptyline plus 5 mg/kg entrectinib *(right)*. Below corresponding time–activity curves (TACs) for the radioactivity tumor uptake profiles and the semi-quantified data depicting the single SUV_mean_ at 60 min post-injection. **B** Effect on BAT uptake of [^18^F]TRACK. Representative PET images as maximum intensity projections (MIP) at 60 min post-injection of 5–7 MBq [^18^F]TRACK into wild-type control mice. Control image *(left)*, after injection of 15 mg/kg amitriptyline *(middle)* and after combined injection of 15 mg/kg amitriptyline plus 5 mg/kg entrectinib *(right)*. Below corresponding time–activity curves (TACs) for the radioactivity tumor uptake profiles and the semi-quantified data depicting the single SUV_mean_ at 60 min post-injection. All graphical data are shown as mean ± SEM from 3 to 5 experiments

In a final experimental setup, effects of the pan-Trk inhibitor entrectinib were also tested in KM12 tumor-bearing mice pre-treated with 15 mg/kg amitriptyline before to analyze if there would be still a functional blocking effect in peripheral tissue. Indeed, dynamic PET experiments revealed that in KM12 tissue, the addition of 5 mg/kg of entrectinib reduced [^18^F]TRACK uptake slightly by ~ 8% from SUV_60min_ from 0.53 ± 0.01 (*n* = 5) to 0.49 ± 0.05 (*n* = 3; *p* > 0.05; Fig. 9A) and in brown adipose tissue by ~ 14% from SUV_60min_ from 1.56 ± 0.08 (*n* = 5) to 1.37 ± 0.12 (*n* = 3; *p* > 0.05; Fig. 9B), respectively. Although these effects were small and did not satisfy the criteria for statistical significance, they fit the observable pattern.

## Discussion

The present study revealed the following main results: (i) Trk inhibitor [^18^F]TRACK is taken up into TrkA expressing human KM12 colon cancer cells, and this uptake can be inhibited by pan-Trk inhibitor entrectinib; (ii) [^18^F]TRACK is also taken up into KM12 tumor tissue as well as brown adipose tissue (BAT) in vivo which can also be blocked by entrectinib; and (iii) amitriptyline acts as a functional Trk agonist and increases uptake of [^18^F]TRACK in peripheral target tissue.

Trk expression and function have been mainly studied in the neuronal system as well as for neurological disorders and neurodegenerative diseases [15–19]. In addition, Trk fusion protein expression and activation promoting cell proliferation have been found in many cancers [1]. However, based on their rarity, variety and clinical sample detection limits to date, functional effects, and importance in cancers are not fully understood yet [48]. In 2018 and 2019, first-generation Trk inhibitors, larotrectinib and entrectinib, were introduced into the clinic for targeted therapy effectively blocking the kinase domain activity [49]. Next-generation Trk inhibitors, selitrectinib and repotrectinib, are currently undergoing clinical development to overcome therapy resistance [50]. Clinical data have shown that specific therapy with first-generation Trk inhibitors achieves high response rates independent of tumor histology, age, or *NTRK* fusion type [51]. Patient selection for Trk therapy may become a key step for personalized medicine and treatment decisions. As current clinical testing for *NTRKs* is still based on invasive biopsy sampling or costly novel bioassays such as NGS [48], access to noninvasive imaging methods such as PET would tremendously facilitate therapy decision-making processes in the clinical setting. Even with the limitation of Trk protein expression in muscle tissue [52] which would lead to a certain increased background uptake, overexpression of Trk fusion proteins in certain cancer types could contribute to valuable diagnostic PET information for a specific patient population to support the therapeutic decision and to select only eligible patients for a Trk inhibitor therapy.

After numerous imaging probe developments [37], [^18^F]TRACK has been identified as an optimized ^18^F-labeled Trk targeting PET radiotracer [43, 44]. So far, [^18^F]TRACK has been mainly evaluated for its binding to Trk in the brain. While it has shown substantial brain uptake in preclinical studies in murine, monkey, and human brain [43, 44], in vivo proof of target specificity remains challenging.

In the present study, inhibition of [^18^F]TRACK cellular uptake into KM12 cells using pan-Trk inhibitor entrectinib was successful. KM12 represents a human colon cancer cell line expressing TrkA and more specifically tropomyosin 3-TrkA (*TPM3-NTRK1*) fusion protein [14, 53], since non-radiolabeled TRACK and entrectinib resulted in similar IC_50_ values in the nanomolar range when competing with [^18^F]TRACK binding in KM12 cells. These results serve as a strong functional proof of target in this colon cancer model in vitro. In enzymatic assays, entrectinib has shown IC_50_ values of 1–5 nM against isolated TrkA/B/C [54]. TRACK resulted in an IC_50_ of 0.3–4.2 nM IC_50_ for human TrkA/B/C measured in an ATP-based enzymatic assay [43]. However, uptake into KM12 tumors in vivo was like muscle tissue (SUV_60min_ 0.43 vs. 0.46). This could be attributed to the muscle expression of Trk specifically TrkB as known from the literature [52, 55]. Interestingly, uptake profiles over time resulted in a later slight washout from muscle tissue, while uptake into tumor tissue continued to rise even after 2 h. resulting in a T/M ratio of 1.1.

PET imaging experiments also revealed uptake into brown adipose tissue (BAT) in mice reaching significantly higher uptake levels (SUV_60min_ > 1) versus KM12 tumor tissue. In the literature, adipose tissue has been shown to express TrkB mRNA [56] and TrkB protein in bovine adipose tissue of lactating cows [57]. The neuronal BDNF/TrkB axis and its function in adipose tissue play an important role for metabolic regulation and its influence on food intake and obesity. Therefore, it seems not surprising to detect uptake of [^18^F]TRACK in murine BAT.

TrkB protein expression in brown and white adipose tissue as well as KM12 tumor samples was confirmed using Western blotting and immunohistochemistry and compared to TrkB expression in the whole mouse brain. Experimental analysis revealed that TrkB expression in the brain is high and ubiquitous. Immunohistochemistry also showed somewhat higher expression of TrkA over TrkB in the mouse brain. In contrast, expression of TrkB was stronger than TrkA on the membrane surface of fat cells analyzed from murine BAT samples confirming previous observations in lactating cows [57]. However, present analysis of KM12 tissue with immunohistochemistry did not reveal substantial expression of both TrkA and TrkB. TrkA was only found in small inclusions in the tumor tissue confirming only a somewhat weak expression although KM12 cells are known to strongly express TrkA [58] although as the fusion protein TPM3-NTRK1 [59]. It is possible that the antibody used for western blot and immunohistochemistry experiments in the present study was not able to detect the fusion protein.

The present functional in vivo analysis using radiotracer [^18^F]TRACK supports the fact of lower protein expression and functional availability in KM12 tumors versus higher expression in BAT. To confirm specific targeting of [^18^F]TRACK to Trk (mainly TrkB) in BAT, in vivo blocking studies were performed with non-radioactive TRACK reference compound for self-blocking and pan-Trk inhibitor entrectinib. With both compounds, similar blocking effects on the radiotracer uptake were observed at 30 min p.i. (minus 23% with TRACK and 18% with entrectinib). It must be added that intracellular targets are often difficult to engage experimentally with in vivo blocking PET experiments. This is further based on the necessary higher lipophilicity of the PET tracer compound to reach its intracellular target. We have made similar observations before when analyzing specific binding of radiotracers to intracellular COX-2. While inhibition with selective COX-2 inhibitor, celecoxib, revealed only 24% blocking effect from PET experiments using [^18^F]Pyricoxib, ex vivo biodistribution showed a 50% inhibition [60]. Based on that, blocking effects with TRACK and entrectinib were observed within the same order of magnitude. However, this effect was not persistent and at 60 min p.i., no blocking was detectible indicating reversible effects over time. Based on the reversibility of the observed effects, it is difficult to conclude that these in vivo experiments using standard Trk inhibitors and therefore antagonists binding to the intracellular kinase domain would serve as proof of target in vivo although they show clear blocking effects in KM12 cells in vitro*.*

Consequently, an alternative approach using an agonist on Trk receptors might provide a way to prove specific targeting of TrkB via radiotracer [^18^F]TRACK in vivo. A basic analysis from 2009 revealed that the classic tricyclic antidepressant amitriptyline directly binds to the extracellular domain of both TrkA and TrkB triggering their dimerization and activation [47]. It was found that amitriptyline is in fact acting as an agonist on both TrkA and B in neuronal cells and that inhibition of TrkA eliminated its neuroprotective effect without impairing its antidepressant activity. Subsequent follow-up studies found that amitriptyline induces neurite outgrowth in rat primary cortical neurons through Trk receptor and MAP kinase activation [61] and that similarly to neurotrophins amitriptyline also acts as a Trk receptor agonist on the regeneration of afferent cochlear synapses in the inner ear [62]. Using amitriptyline in the present study, overall uptake levels of [^18^F]TRACK in KM12 tumor tissue and in BAT could be significantly increased by 21 and 23% indicating that ligand activation of TrkA and TrkB in both tissues resulted in increased binding of [^18^F]TRACK at the intracellular kinase binding site as well. For comparison, radiotracer uptake in the whole brain was increased by only 13%, which did not reach statistical significance, though the trend confirmed the observations in the peripheral tumor and BAT tissue. Interestingly, [^18^F]TRACK uptake into muscle tissue was also elevated by 15%, however, only after 30 min and diminished after 60 min which is different from the effect in tumor and BAT tissue attributing maybe to different intracellular ATP levels to compete for binding with [^18^F]TRACK or different dimerization forms of the oncogene Trk versus the muscular Trk. It is known that muscle tissue expresses Trk [55] and specifically TrkB and that BDNF mimetics such as 7,8-di-hydroxyflavone [52] or other antidepressant drugs such as fluoxetine can activate muscular TrkB by direct binding to the receptor [63]. Taken together, tricyclic antidepressant amitriptyline also acts as a Trk agonist in peripheral target tissue by activating Trk receptors resulting in an uptake increase in Trk targeting radiotracer [^18^F]TRACK. In addition, this in vivo experiment also serves as a pharmacodynamic proof-of-target experiment.

Finally, experiments combining agonistic activation of Trk with amitriptyline (binding at the extracellular domain) and Trk inhibition with entrectinib (binding the intracellular kinase domain) revealed that there was a small but also systematic blocking effect detectable at 60 min post-injection of radiotracer [^18^F]TRACK in both KM12 tumor tissue (Δ8%) and BAT (Δ14%). This effect was persistent in the equilibrium phase of the radiotracer’s in vivo distribution which contrasted with the observed reversible effects detected without amitriptyline. This combination experiment may serve as the best functional proof of target for selective target binding of [^18^F]TRACK in vivo and in combination with the in vitro results.

## Conclusion

The present study demonstrated that novel Trk targeting PET radiotracer [^18^F]TRACK binds to TrkA and TrkB in peripheral tissue such as colon KM12 cancer and adipose tissue but also muscle tissue. Besides its use for noninvasive functional brain PET imaging to detect changes in neurodegenerative diseases as analyzed before, the present data also support the potential application as an additional tool for multiple diagnostic tumor imaging to detect the right patients with Trk protein expression in cancers and to select them for specific Trk inhibitor therapy. Additionally, visualization of TrkB in adipose tissue of obese patients may open a new field for imaging Trk involvement in in metabolic disease. While first-in-human PET has been recently performed with [^18^F]TRACK for brain PET imaging and current clinical studies are undergoing in Alzheimer disease patients, the present dataset may also support a clinical translation for cancer and metabolic disease as well.

## Supplementary Information

**Additional file1**. **Fig. S1**. Brain uptake of [^18^F]TRACK in control and 50% TrkB knockout mice from dynamic PET experiments. Representative images at 30 min post injection and time-activity curves over 120 min time course; **Fig. S2**. TrkA and TrkB protein expression in mouse brain. Immunohistochemical staining in slices from a KM12 tumor bearing athymic mouse and a control wild-type B619SF2/J mouse; **Fig. S3**. Uptake of [^18^F]TRACK into brown adipose tissue (BAT). Time activity curves for BAT tissue in control wild-type B6129SF2/J mice and B6129S2-Ntrk2tm1Bbd/J 50% TrkB knockout mice over 120 min time course. **Fig. S4**. Muscle uptake of [^18^F]TRACK in KM12 tumor bearing mice. Effect of 15 mg/kg amitriptyline on the time-activity curve over 60 min time course. **Fig. S5**. Original full gel for the protein expression of TrkA and TrkB. Data are shown in samples from KM12 tumor tissue, brown adipose tissue (BAT), white adipose tissue (AT) and the whole brain from KM12-tumor bearing mice, control wild-type B6129SF2/J mice and B6129S2-Ntrk2tm1Bbd/J 50% TrkB knockout mice.

## Data Availability

Experimental data are available from the corresponding authors on reasonable request.

## Abbreviations

BAT: Brown adipose tissue
BDNF: Brain-derived neurotrophic factor
FISH: Fluorescence in situ hybridization
MAP: Maximum a posteriori
NGF: Nerve growth factor
NSG: Next-generation sequencing
OSEM: Ordered subset expectation maximization
PET: Positron emission tomography
ROI: Region of interest
SUV_mean_: Mean standardized uptake value
TAC: Time–activity curve
Trk: Tropomyosin receptor kinase
T/M: Tumor-to-muscle ratio
WB: Western Blot

## References

[1] Vaishnavi A, Le AT, Doebele RC (2015). TRKing down an old oncogene in a new era of targeted therapy. Cancer Discov.

[2] Amatu A, Sartore-Bianchi A, Bencardino K, Pizzutilo EG, Tosi F, Siena S (2019). Tropomyosin receptor kinase (TRK) biology and the role of NTRK gene fusions in cancer. Ann Oncol.

[3] Lamballe F, Klein R, Barbacid M (1991). TrkC, a new member of the trk family of tyrosine protein kinases, is a receptor for neurotrophin-3. Cell.

[4] Kaplan DR, Hempstead BL, Martin-Zanca D, Chao MV, Parada LF (1991). The trk protooncogene product: a signal transducing receptor for nerve growth factor. Science.

[5] Kaplan DR, Martin-Zanca D, Parada LF (1991). Tyrosine phosphorylation and tyrosine kinase activity of the trk proto-oncogene product induced by NGF. Nature.

[6] Barbacid MJ (1994). The Trk family of neurotrophin receptors. Neurobiol.

[7] Malcangio M, Lessmann V (2003). A common thread for pain and memory synapses? Brain-derived neurotrophic factor and TrkB receptors. Trends Pharmacol Sci.

[8] Binder DK, Scharfman HE (2004). Brain-derived neurotrophic factor. Growth Factors.

[9] Bothwell M (2014). NGF, BDNF, NT3, and NT4. Handb Exp Pharmacol.

[10] Reichardt LF (2006). Neurotrophin-regulated signalling pathways. Philos Trans R Soc Lond B Biol Sci.

[11] Bertrand T (2017). Crystal structures of neurotrophin receptors kinase domain. Vitam Horm.

[12] Barbacid M, Lamballe F, Pulido D, Klein R (1991). The Trk family of tyrosine protein kinase receptors. Biochim Biophys Acta.

[13] Huang EJ, Reichardt LF (2003). Trk receptors: roles in neuronal signal transduction. Annu Rev Biochem.

[14] Vaishnavi A, Capelletti M, Le AT, Kako S, Butaney M, Ercan D (2013). Oncogenic and drug-sensitive NTRK1 rearrangements in lung cancer. Nat Med.

[15] Connor B, Dragunow M (1998). The role of neuronal growth factors in neurodegenerative disorders of the human brain. Brain Res Rev.

[16] Zhang F, Kang Z, Li W, Xiao Z, Zhou X (2012). Roles of brain-derived neurotrophic factor/tropomyosin-related kinase B (BDNF/TrkB) signalling in Alzheimer’s disease. J Clin Neurosci.

[17] Reinhart V, Bove SE, Volfson D, Lewis DA, Kleiman RJ, Lanz TA (2015). Evaluation of TrkB and BDNF transcripts in prefrontal cortex, hippocampus, and striatum from subjects with schizophrenia, bipolar disorder, and major depressive disorder. Neurobiol Dis.

[18] Song J-H, Yu J-T, Tan L (2015). Brain-derived neurotrophic factor in Alzheimer’s disease: risk, mechanisms, and therapy. Mol Neurobiol.

[19] Meldolesi J (2017). Neurotrophin receptors in the pathogenesis, diagnosis and therapy of neurodegenerative diseases. Pharmacol Res.

[20] Ricciuti B, Genova C, Crinò L, Libra M, Leonardi GC (2019). Antitumor activity of larotrectinib in tumors harboring NTRK gene fusions: a short review on the current evidence. Onco Targets Ther.

[21] Rohrberg KS, Lassen U (2021). Detecting and targeting NTRK fusions in cancer in the era of tumor agnostic oncology. Drugs.

[22] Gatalica Z, Xiu J, Swensen J, Vranic S (2019). Molecular characterization of cancers with NTRK gene fusions. Mod Pathol.

[23] Koizumi H, Morita M, Mikami S, Shibayama E, Uchikoshi T (1998). Immunohistochemical analysis of TrkA neurotrophin receptor expression in human non-neuronal carcinomas. Pathol Int.

[24] Roviello G, D'Angelo A, Sciortino M, Mini E, Nobili S, De Logu F (2020). TRK fusion positive cancers: from first clinical data of a TRK inhibitor to future directions. Crit Rev Oncol Hematol.

[25] Stenzinger A, van Tilburg CM, Tabatabai G, Länger F, Graf N, Griesinger F (2021). Diagnosis and therapy of tumors with NTRK gene fusion. Pathologe.

[26] Federman N, McDermott R (2019). Larotrectinib, a highly selective tropomyosin receptor kinase (TRK) inhibitor for the treatment of TRK fusion cancer. Expert Rev Clin Pharmacol.

[27] Liu D, Offin M, Harnicar S, Li BT, Drilon A (2018). Entrectinib: an orally available, selective tyrosine kinase inhibitor for the treatment of NTRK, ROS1, and ALK fusion-positive solid tumors. Ther Clin Risk Manag.

[28] Albert CM, Davis JL, Federman N, Casanova M, Laetsch TW (2019). TRK fusion cancers in children: a clinical review and recommendations for screening. J Clin Oncol.

[29] Cocco E, Scaltriti M, Drilon A (2018). NTRK fusion-positive cancers and TRK inhibitor therapy. Nat Rev Clin Oncol.

[30] Hsiao SJ, Zehir A, Sireci AN, Aisner DL (2019). Detection of tumor NTRK gene fusions to identify patients who may benefit from tyrosine kinase (TRK) inhibitor therapy. J Mol Diagn.

[31] Kirchner M, Glade J, Lehmann U, Merkelbach-Bruse S, Hummel M, Lehmann A (2020). NTRK testing: first results of the QuiP-EQA scheme and a comprehensive map of NTRK fusion variants and their diagnostic coverage by targeted RNA-based NGS assays. Genes Chromosomes Cancer.

[32] Marchiò C, Scaltriti M, Ladanyi M, Iafrate AJ, Bibeau F, Dietel M (2019). ESMO recommendations on the standard methods to detect NTRK fusions in daily practice and clinical research. Ann Oncol.

[33] Penault-Llorca F, Rudzinski ER, Sepulveda AR (2019). Testing algorithm for identification of patients with TRK fusion cancer. J Clin Pathol.

[34] Pfarr N, Kirchner M, Lehmann U, Leichsenring J, Merkelbach-Bruse S, Glade J (2020). Testing NTRK testing: wet-lab and in silico comparison of RNA-based targeted sequencing assays. Genes Chromosomes Cancer.

[35] Solomon JP, Hechtman JF (2019). Detection of NTRK fusions: merits and limitations of current diagnosticplatforms. Cancer Res.

[36] Solomon JP, Linkov I, Rosado A, Mullaney K, Rosen EY, Frosina D (2020). NTRK fusion detection acrossmultiple assays and 33,997 cases: diagnostic implications and pitfalls. Mod Pathol.

[37] Schirrmacher R, Bernard-Gauthier V, Jaworski C, Wangler C, Wangler B, Bailey JJ, Dierckx RAJO, Otte A, deVries EFJ, van Waarde A (2021). Toward imaging tropomyosin receptor kinase (Trk) with positron emission tomography. PET and SPECT of neurobiological systems.

[38] Bernard-Gauthier V, Aliaga A, Boudjemeline M, Hopewell R, Kostikov A, Rosa-Neto P (2015). Syntheses and evaluation of Carbon-11- and Fluorine-18-radiolabeled pan-tropomyosin receptor kinase (Trk) inhibitors: exploration of the 4-Aza-2-oxindole scaffold as Trk PET imaging agents. ACS Chem Neurosci.

[39] Bernard-Gauthier V, Schirrmacher R (2014). 5-(4-((4-[18F]fluorobenzyl)oxy)-3-methoxy-benzyl)pyrimidine-2,4-diamine: a selective dual inhibitor for potential PET imaging of Trk/CSF-1R. Bioorg Med Chem Lett.

[40] Bernard-Gauthier V, Mahringer A, Vesnaver M, Fricker G, Schirrmacher R (2017). Design and synthesis of a fluorinated quinazoline-based type-II Trk inhibitor as a scaffold for PET radiotracer development. Bioorg Med Chem Lett.

[41] Bernard-Gauthier V, Bailey JJ, Aliaga A, Kostikov A, Rosa-Neto P, Wuest M (2015). Development of subnanomolar radiofluorinated (2-pyrrolidin-1-yl)imidazo[1,2-b]pyridazine pan-Trk inhibitors as candidate PET imaging probes. Med Chem Comm.

[42] Bernard-Gauthier V, Bailey JJ, Mossine AV, Lindner S, Vomacka L, Aliaga A (2017). A Kinome-wide selective radiolabeled TrkB/C inhibitor for in vitro and in vivo neuroimaging: synthesis, preclinical evaluation, and first-in-human. J Med Chem.

[43] Bernard-Gauthier V, Mossine AV, Mahringer A, Aliaga A, Bailey JJ, Shao X (2018). Identification of [18F]TRACK, a Fluorine-18-labeled tropomyosin receptor kinase (Trk) inhibitor for PET imaging. J Med Chem.

[44] Bailey JJ, Kaiser L, Lindner S, Wüst M, Thiel A, Soucy JP (2019). First-in-human brain imaging of [18F]TRACK, a PET tracer for tropomyosin receptor kinases. ACS Chem Neurosci.

[45] Zischler J, Kolks N, Modemann D, Neumaier B, Zlatopolskiy BD (2017). Alcohol-enhanced Cu-mediated radiofluorination. Chemistry.

[46] Varghese F, Bukhari AB, Malhotra R, De A (2014). IHC profiler: an open source plugin for the quantitative evaluation and automated scoring of immunohistochemistry images of human tissue samples. PLoS ONE.

[47] Jang SW, Liu X, Chan CB, Weinshenker D, Hall RA, Xiao G (2009). Amitriptyline is a TrkA and TrkB receptor agonist that promotes TrkA/TrkB heterodimerization and has potent neurotrophic activity. Chem Biol.

[48] Kojadinovic A, Laderian B, Mundi PS (2021). Targeting TRK: A fast-tracked application of precision oncology and future directions. Crit Rev Oncol Hematol.

[49] Laetsch TW, Hong DS (2021). Tropomyosin receptor kinase inhibitors for the treatment of TRK fusion cancer. Clin Cancer Res.

[50] Drilon A, Nagasubramanian R, Blake JF, Ku N, Tuch BB, Ebata K (2017). A next-generation TRK kinase inhibitor overcomes acquired resistance to prior TRK kinase inhibition in patients with TRK fusion-positive solid tumors. Cancer Discov.

[51] Drilon A (2019). TRK inhibitors in TRK fusion-positive cancers. Ann Oncol.

[52] Chan CB, Tse MC, Liu X, Zhang S, Schmidt R, Otten R (2015). Activation of muscular TrkB by its small molecular agonist 7,8-dihydroxyflavone sex-dependently regulates energy metabolism in diet-induced obese mice. Chem Biol.

[53] Tatematsu T, Sasaki H, Shimizu S, Okuda K, Shitara M, Hikosaka Y (2014). Investigation of neurotrophic tyrosine kinase receptor 1 fusions and neurotrophic tyrosine kinase receptor family expression in non-small-cell lung cancer and sensitivity to AZD7451 in vitro. Mol Clin Oncol.

[54] Ardini E, Menichincheri M, Banfi P, Bosotti R, De Ponti C, Pulci R (2016). Entrectinib, a Pan-TRK, ROS1, and ALK inhibitor with activity in multiple molecularly defined cancer indications. Mol Cancer Ther.

[55] Reinach FC, MacLeod AR (1986). Tissue-specific expression of the human tropomyosin gene involved in the generation of the trk oncogene. Nature.

[56] Nakagomi A, Okada S, Yokoyama M, Yoshida Y, Shimizu I, Miki T (2015). Role of the central nervous system and adipose tissue BDNF/TrkB axes in metabolic regulation. NPJ Aging Mech Dis.

[57] Colitti M, Loor JJ, Stefanon B (2015). Expression of NGF, BDNF and their receptors in subcutaneous adipose tissue of lactating cows. Res Vet Sci.

[58] Park DY, Choi C, Shin E, Lee JH, Kwon CH, Jo HJ (2016). NTRK1 fusions for the therapeutic intervention of Korean patients with colon cancer. Oncotarget.

[59] Ardini E, Bosotti R, Borgia AL, De Ponti C, Somaschini A, Cammarota R (2014). The TPM3-NTRK1 rearrangement is a recurring event in colorectal carcinoma and is associated with tumor sensitivity to TRKA kinase inhibition. Mol Oncol.

[60] Tietz O, Wuest M, Marshall A, Glubrecht D, Hamann I, Wang M (2016). PET imaging of cyclooxygenase-2 (COX-2) in a pre-clinical colorectal cancer model. EJNMMI Res.

[61] O'Neill E, Kwok B, Day JS, Connor TJ, Harkin A (2016). Amitriptyline protects against TNF-α-induced atrophy and reduction in synaptic markers via a Trk-dependent mechanism. Pharmacol Res Perspect.

[62] Fernandez KA, Watabe T, Tong M, Meng X, Tani K, Kujawa SG (2021). Trk agonist drugs rescue noise-induced hidden hearing loss. JCI Insight.

[63] Casarotto PC, Girych M, Fred SM, Kovaleva V, Moliner R, Enkavi G (2021). Antidepressant drugs act by directly binding to TRKB neurotrophin receptors. Cell.

