# Supplementary material for: Toward in vivo proof of binding of 18F-labeled inhibitor [18F]TRACK to peripheral tropomyosin receptor kinases
Source: EJNMMI Res. 2022 Jul 30;12:46. doi: 10.1186/s13550-022-00915-w (PMC9339071; doi:10.1186/s13550-022-00915-w)
Supplement: Supplementary file 1 — Additional file1. Fig. S1. Brain uptake of [18F]TRACK in control and 50% TrkB knockout mice from dynamic PET experiments. Representative images at 30 min post injection and time-activity curves over 120 min time course; Fig. S2. TrkA and TrkB protein expression in mouse brain. Immunohistochemical staining in slices from a KM12 tumor bearing athymic mouse and a control wild-type B619SF2/J mouse; Fig. S3. Uptake of [18F]TRACK into brown adipose tissue (BAT). Time activity curves for BAT tissue in control wild-type B6129SF2/J mice and B6129S2-Ntrk2tm1Bbd/J 50% TrkB knockout mice over 120 min time course. Fig. S4. Muscle uptake of [18F]TRACK in KM12 tumor bearing mice. Effect of 15 mg/kg amitriptyline on the time-activity curve over 60 min time course. Fig. S5. Original full gel for the protein expression of TrkA and TrkB. Data are shown in samples from KM12 tumor tissue, brown adipose tissue (BAT), white adipose tissue (AT) and the whole brain from KM12-tumor bearing mice, control wild-type B6129SF2/J mice and B6129S2-Ntrk2tm1Bbd/J 50% TrkB knockout mice. [file 13550_2022_915_MOESM1_ESM.docx]

**Supplementary**

**Towards In Vivo Proof-of-Binding of ^18^F-labeled Inhibitor [^18^F]TRACK to Peripheral Tropomyosin Receptor Kinases**

Melinda Wuest^1*^, Justin J. Bailey^1^, Jennifer Dufour^1^, Darryl Glubrecht^1^, Vanessa Omana^2^, Tom H. Johnston^3^, Jonathan M. Brotchie^3^, Ralf Schirrmacher^1*^

*^1^Department of Oncology and Cross Cancer Institute, University of Alberta, Edmonton, Alberta, Canada*

*^2^The Neuro - Montreal Neurological Institute-Hospital, McGill University, Montreal, Quebec, Canada*

*^3^Krembil Research Institute, University Health Network, Toronto, Ontario, Canada*

*^4^Atuka Inc., Toronto, Ontario, Canada.*

[^18^F]TRACK

**A)**


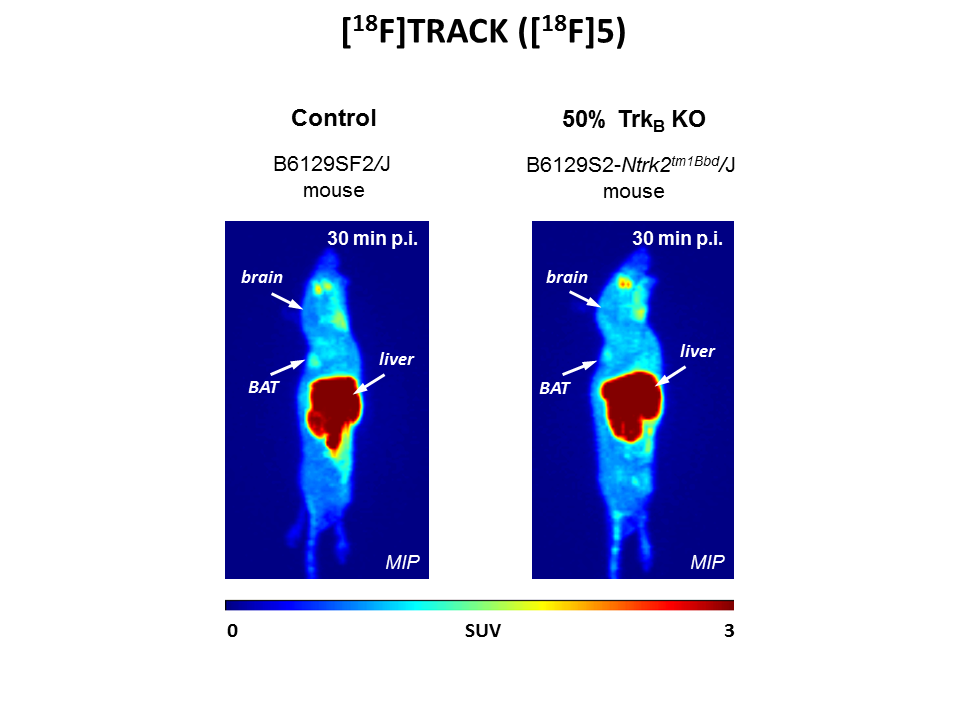


**B)**

Figure S1: A) Representative PET images as maximum intensity projections (MIP) after injection of [^18^F]TRACK at 30 min post injection. Left: B6129SF2/J control mouse and Right: B6129S2-*Ntrk2*^tm1Bbd^/J 50% TrkB knock out mouse. B) Time-activity curves for the radioactivity distribution in the whole brain over the time frame of 2 hours. Data are shown as SUV mean values from 3 experiments each.


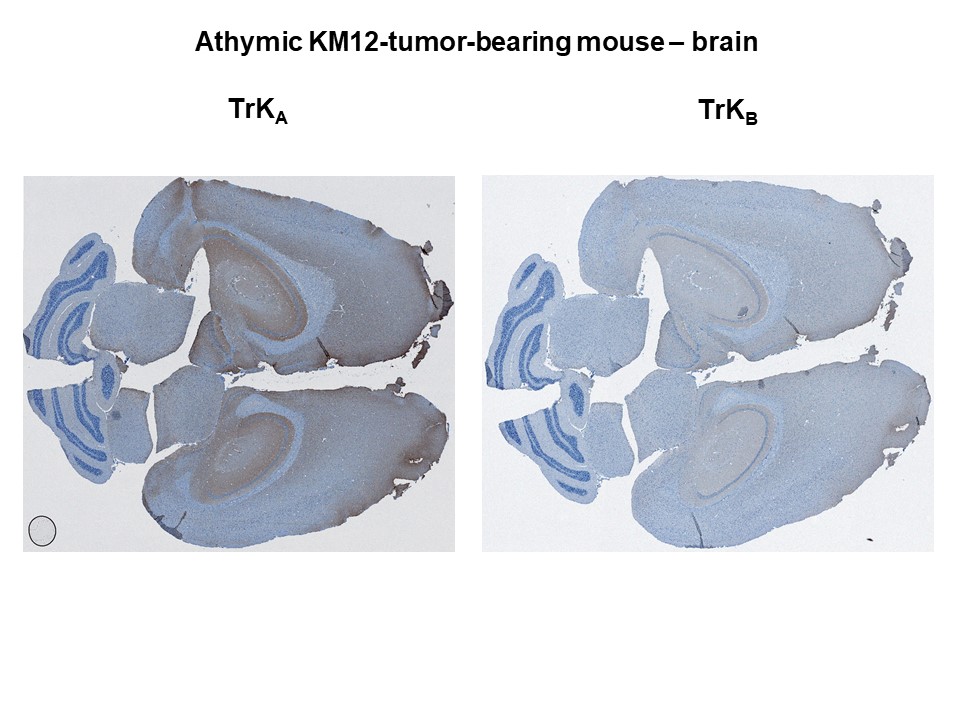

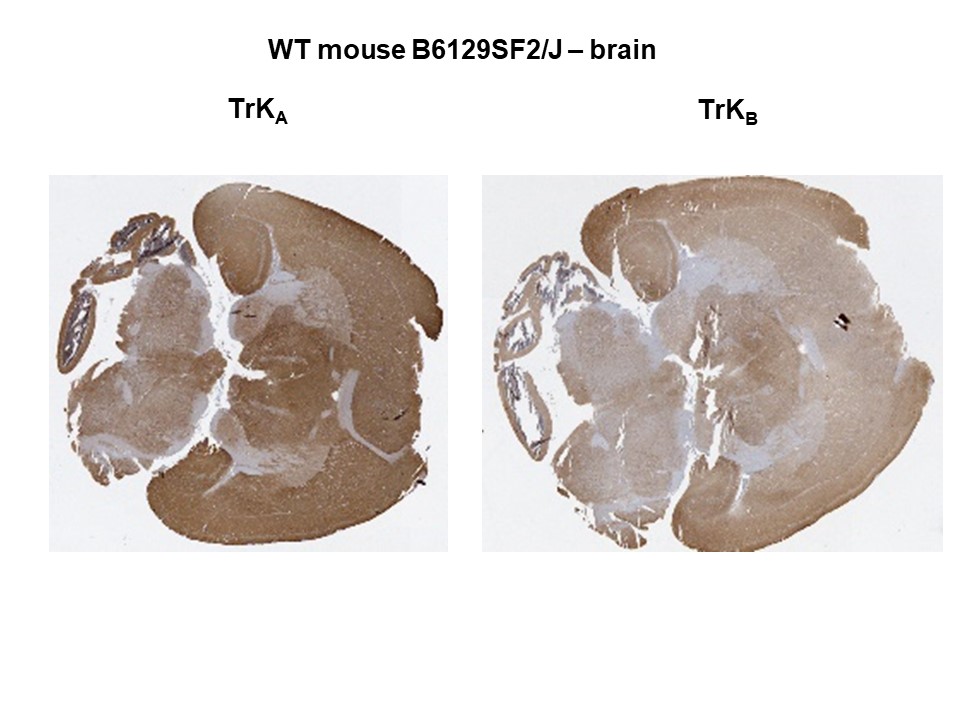


Figure S2: Immunohistochemical staining of TrkA and TrkB in the whole brain of an athymic KM12-tumor-bearing mouse (top) and a wild type (WT) control B6129SF2/J mouse (bottom). Pictures were taken using a 5x objective.

**[^18^F]TRACK**

Figure S3: Time-activity curves for the radioactivity uptake into brown adipose tissue (BAT) of WT B6129SF2/J control mice and B6129S2-*Ntrk2*^tm1Bbd^/J 50% TrkB knock out mice over the time frame of 2 hours. Data are shown as SUV mean values from 3 experiments each.

Figure S4: Time-activity curves for the radioactivity uptake profile in mouse muscle tissue of KM12-tumor bearing athymic nude mice over the time frame of 60 min after injection of [^18^F]TRACK. Data were collected in the absence and presence of 15 mg/kg amitriptyline (pre-dosed i.p. 4 hours before radiotracer injection) Data are shown as SUV mean values from 5 experiments each.


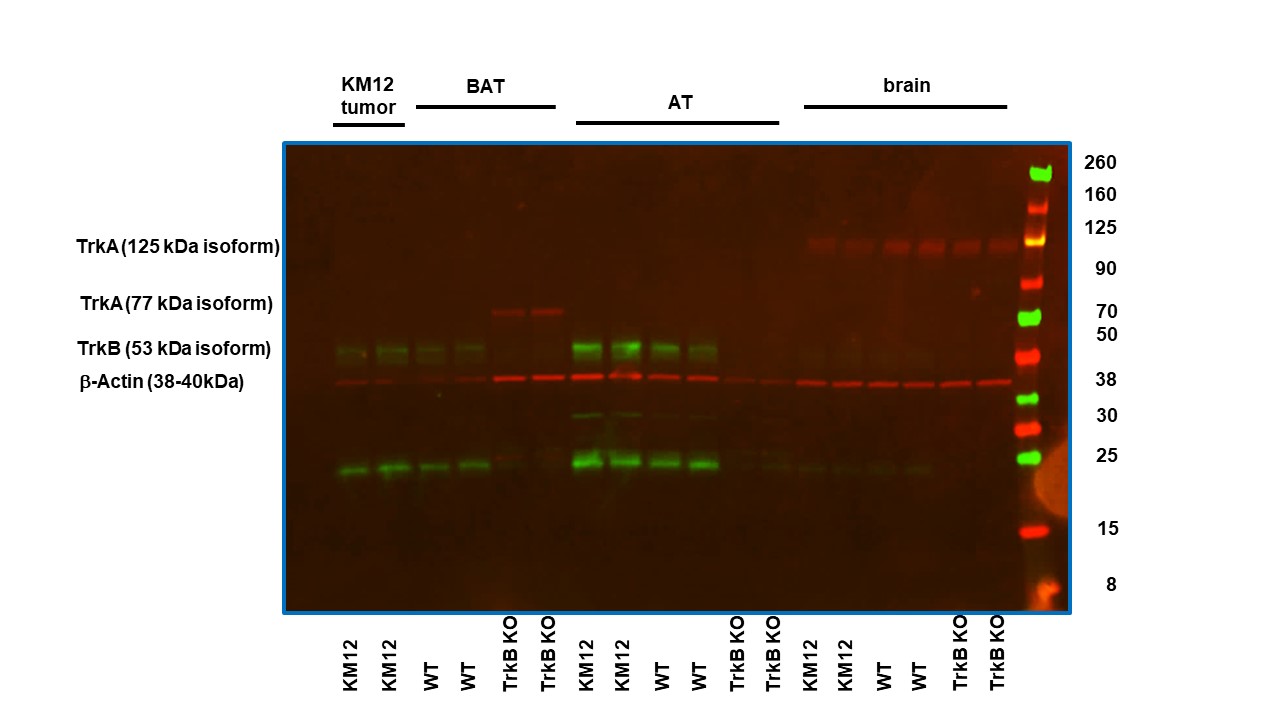


Figure S5: Original full gel for the protein expression of TrkA and TrkB in samples from KM12 tumor tissue, brown adipose tissue (BAT), white adipose tissue (AT) and the whole brain from KM12-tumor bearing mice, control wild type (WT) and TrkB 50% KO mice from combined red (700 nm) and green (800 nm) light wavelength analysis showing using antibodies for TrkA and TrkB and β-actin as housekeeping gene.
